# *Encephalitozoon cuniculi* infection in farmed rabbits in Egypt

**DOI:** 10.1186/s13028-020-0509-6

**Published:** 2020-02-22

**Authors:** Eman Anter Morsy, Heba Mohammed Salem, Marwa Salah Khattab, Dalia Anwar Hamza, Mai Mohammed Abuowarda

**Affiliations:** 1 Department of Poultry Diseases, Faculty of Veterinary Medicine, Cairo University, PO Box 12211, Giza, Egypt; 2 Department of Pathology, Faculty of Veterinary Medicine, Cairo University, PO Box 12211, Giza, Egypt; 3 Department of Zoonoses, Faculty of Veterinary Medicine, Cairo University, PO Box 12211, Giza, Egypt; 4 Department of Parasitology, Faculty of Veterinary Medicine, Cairo University, PO Box 12211, Giza, Egypt

**Keywords:** *Encephalitozoon cuniculi*, Histopathology, Incidence, Polymerase chain reaction, Rabbit, Transmission electron microscopy

## Abstract

**Background:**

*Encephalitozoon cuniculi* is an important microsporidian parasite with zoonotic potential. The present study highlights the impact of encephalitozoonosis on rabbit health in Egypt. Three rabbit farms in Giza, with a total of 16,400 rabbits were investigated due to occurrence of rabbits displaying clinical signs consistent with encephalitozoonosis.

**Results:**

Clinical signs observed during a 4 months observation period in 2018 included vestibular disease, paresis, limb paralysis, cataracts, phacoclastic uveitis, frequent urination, marked decrease in body weight and in some pregnant females, also repeated abortions. The total morbidity rates in adult and young rabbits were 76.7% and 81.5%, respectively. The highest mortality rate was recorded in offspring (12.3%), followed by dams (5.6%), and the lowest recorded mortality rate was in males (0.04%). Post-mortem findings included enteritis, pale enlarged kidneys, congested leptomeninges, focal brain necrosis, and endometrial congestion. Histopathological examination revealed nonsuppurative meningoencephalitis and glial nodules with central necrosis in the brain, vacuolation and necrosis of renal tubular epithelium, and corneal ulceration and ruptured lens capsule with fragmentation of lenticular fibres. *E. cuniculi* were observed in the brain, retinal ganglion cells, kidneys, and liver. Transmission electron microscopy examination revealed the presence of different developmental stages of *E. cuniculi* in the brain and kidney. Presence of *E. cuniculi* was confirmed by conventional polymerase chain reaction using a universal 16S gene for *Encephalitozoon* spp. followed by sequencing and sequence analysis.

**Conclusions:**

The presence of *E. cuniculi* in rabbits was confirmed at three farms in Egypt. Nervous signs and ocular lesions were the most predominant findings in these farms.

## Background

*Encephalitozoon cuniculi* is an obligate intracellular spore-forming protozoan parasite belonging to the phylum Microsporidia, which contains approximately 1400 species distributed into about 200 genera. *E. cuniculi* can infect almost all invertebrates and vertebrates, as well as some protists [1]. *E. cuniculi,* which was first identified in 1922 in a colony of research rabbits in the USA, is now recognized as a common pathogen and a significant cause of disease among pet rabbits [2–4]. *E. cuniculi* has a zoonotic potential, especially for immunocompromised adults and for children [5]. Therefore, *E. cuniculi* has received increased attention [6], and hygienic precautions should be undertaken when humans are in contact with rabbits or rabbit products [7].

The life cycle of *E. cuniculi* takes 3 to 5 weeks to complete [8]. Hosts are infected by ingestion or inhalation of spores or by transplacental transmission [9]. After ingestion, the spores invade enterocytes and then enter the bloodstream or the lymphatic system, possibly through Peyer's patches or interepithelial lymphocytes, and thereafter reach organs such as the brain, kidney and liver. Spores can also transmigrate after having been ingested by phagocytes present in the intestinal mucosa [10]. Infected rabbits develop serum antibodies generally within 3 weeks post-exposure and excrete *E. cuniculi* by 6 weeks [11]. The spores are excreted through urine and faeces; hence, transmission generally takes place after the ingestion of water or food contaminated with infective spores [5].

*Encephalitozoon cuniculi* infection of the central nervous system is associated with development of vestibular disease, which is characterized by torticollis, ataxia, paresis, nystagmus, seizures, and longitudinal rolling and the animal often becomes recumbent [12, 13]. In addition to nervous signs, infected rabbits develop eye lesions and chronic renal failure [14]. In the eye, inflammation of the anterior lens capsule can lead to its spontaneous rupture. Moreover, phacoclastic uveitis develops because of perilenticular fibroplasia together with lens capsule rupture [15, 16]. Kidney lesions can be observed within 4 weeks, and brain lesions within 8 weeks after infection [17]. Microscopic lesions in the brain and kidney, consist of focal non-suppurative granulomatous meningoencephalitis and focal to segmental interstitial nephritis with varying degrees of fibrosis [18].

Transmission electron microscopy (TEM) can be used to confirm encephalitozoonosis by detecting the polar filament within spores and demonstrating ultrastructural features. TEM, along with newly applied molecular approaches, can contribute to the taxonomic organization of the microsporidia [19, 20]. Polymerase chain reaction (PCR)-based methods that typically utilize primers for amplification of microsporidial rDNA have been commonly utilized to enhance diagnostic sensitivity and specificity; however, they are still not routinely used in diagnostic laboratories [21]. The *E. cuniculi* genome is very small being only 2.9 Mb large and compressed. It harbours 2000 genes that are located within minute intergenic regions (the mean intergenic region is 80 bp) [22]. Analyses of genome diversity have revealed the existence of four genotypes of *E. cuniculi* (EcI, II, III and IV) [23].

Rabbit production recently has become well established in Egypt, but little is known about the exact rabbit numbers. Most of the rabbit population in Egypt is in the hands of smallholders, while the rest belongs to the commercial sector as a good source of protein [24]. Rabbits are also used for medical purposes such as the diagnosis of infectious diseases, production of vaccines and other biological substances of public health and veterinary importance [25]. Parasites are responsible for direct and indirect losses of rabbits that are attributed to acute illness and death, premature slaughter, decreased growth rate, weight loss and late maturity of slaughter stock [26].

In Egypt, *E. cuniculi* was not reported until 2011, when its presence was indicated by serological screening of the domestic rabbit population in northern Egypt, in Behera, Alexandria and Khafr El-Sheikh for *E. cuniculi* and *Toxoplasma gondii* [7]. However morphometric or pathological investigations have not yet been carried out.

The purposes of this study were (1) to investigate encephalitozoonosis in rabbit farms in middle Egypt; (2) to confirm the presence of *E. cuniculi* using pathology and molecular techniques and (3) to describe clinical signs, histopathological changes, morphology features and genetic characteristics of the parasite.

## Methods

### Animals

Three commercial rabbit farms (A–C) in the Giza governorate in middle Egypt, with a total of 16,400 rabbits (4121, 5583 and 6696 rabbits for each of the three farms, respectively) founded the study population. Rabbits on these farms were investigated during a 4 months period in 2018.

Rabbits that exhibited clinical signs suggestive of *E. cuniculi* infection (n = 13,242) underwent a clinical examination. Of these 13,242 rabbits, 2680, 4658 and 5904 rabbits came from farms A, B and C, respectively.

Rabbits of different breeds, including New Zealand, Chinchilla, and Californian*,* were reared in a wire net cage system and fed commercial pellets. Rabbit handling procedures were performed in accordance with the applicable legislation of the Institutional Animal Care and Use Committee of the Faculty of Veterinary Medicine, Cairo University, Egypt (VetCU11112018015).

The examined animals were classified into two age groups, i.e. young and adults. Young rabbits (n = 11,170) were from 0 to 4 months old, whereas adults (n = 2072) were rabbits older than 4 months and up to 1 year old. Of the adult rabbits, 332 were males and 1740 females.

### Clinical examination

The rabbits were visually assessed from a distance and any abnormal behaviour was recorded. Each visual assessment took about 5 min/cage. If abnormal behaviour was observed, the rabbit was taken out of the cage and examined according to [27]. This consisted of inspection of the head, including ears, eyes, nose, mucous membranes and lymph nodes and abdominal palpation of the stomach, small intestines, caecum, colon, kidneys, bladder and the uterus. Finally, the perineum and limbs were examined. Securing of the rabbits was performed by placing them sitting in a compact posture while placing two hands lightly over their shoulders.

### Post mortem examination and sample collection

Necropsy was performed on two subgroups of rabbits: (1) rabbits with severe clinical signs that were euthanized and (2) rabbits that had died shortly before a farm visit. The number of required euthanised rabbits was calculated to 376 by using the Open EPI free software https://www.openepi.com/SampleSize/SSPropor.htm and a confidence level of 95%. However, the owners only allowed euthanasia of 341 rabbits. These were anaesthetized by intramuscular injection of ketamine (30 mg/kg) and xylazine (5 mg/kg) and then euthanized by exsanguination [28]. The group of recently dead rabbits consisted of 830 animals.

Samples for laboratory analyses were taken from 30 of the euthanized rabbits and from 20 of the recently dead rabbits from each farm giving a total number of 150 rabbits. These 150 rabbits were selected randomly from each farm among necropsied rabbits with prominent lesions indicating encephalitozoonosis. The following laboratory examinations were performed: (1) histopathology was performed on 10 rabbits from each of the three farms, (2) TEM was performed on seven rabbits, and (3) PCR was done on all 150 rabbits.

### Histopathological examination

Specimens of the brain, kidney, liver, and eye from the 30 rabbits were fixed in 10% neutral buffered formalin. The samples were then processed, paraffin-embedded, sectioned at 3 µm thickness and stained with haematoxylin and eosin and Giemsa stain [29].

### Transmission electron microscopy examination

Brain and kidney specimens from the seven rabbits were fixed in 2.5% glutaraldehyde, kept for 4 h at 4 °C, thoroughly rinsed overnight at 4 °C in 0.1 M phosphate buffer, fixed in 1% osmium tetraoxide (OsO_4_) for 30 min at 4 °C, then washed in PBS (pH 7.3) for 1 h followed by dehydration in an ethanol gradient (50–90% for 15 min, 100% for 1 h). The samples were then routinely processed and embedded in Epon-Araldite. Semithin sections (0.5 µm) were cut with a glass knife using a microtome (RMC Inc., Tucson, AZ, USA) and collected on copper grids and then stained with 1% toluidine blue for 20 min, washed in distilled water and placed in Permount. The obtained slides were examined and photographed using an Olympus DP20 microscope (Olympus, Tokyo, Japan) [14]. Ultrathin sections were stained for 15 min with a saturated uranyl acetate solution and counterstained in lead citrate for 20 min (thickness 500–700 Angstrom). Grids containing ultrathin sections of the material were analysed and photographed using a JEM-1200 EX II transmission electron microscope (Jeol, Tokyo, Japan). This was performed at the Electron Microscope Unit at the Military Veterinary Hospital in Nasr City, Cairo, Egypt.

### DNA extraction

DNA was extracted from brain, eyeball, liver, and kidney of the 150 rabbits using the DNeasy Blood & Tissue Kit (Qiagen, Düsseldorf, Germany) according to the manufacturer’s instructions.

PCR was performed for the target regions of the small subunit of 16S rRNA of zoonotic microsporidia, including *E. bieneusi, E. intestinalis, E. cuniculi, E. hellem, Vittaforma corneae,* and *Pleistophora* spp. using the forward primer F (5′-CAC CAG GTT GAT TCT GCC TGAC-3′) and reverse primer R (5′-CCT CTC CGG AAC CAA ACC CTG-3′) [30]. These primers are designed to generate amplicons of 260 bp. The amplification was performed using reaction conditions according to [30].

### Sequencing of PCR products and sequence analysis

Among the PCR positive products, 10 samples representing three from each of farm A and B and four from farm C were purified using the QIAquick purification kit (Qiagen) according to the manufacturer’s instructions and sequencing was conducted using the Big Dye Terminator V3.1 Sequencing kit (Applied Biosystems, Waltham, USA) with the forward and reverse primer for 16S rRNA. The obtained 10 nucleotide sequences were aligned with the sequences in GenBank using the NCBI BLAST server to confirm the identity with *E. cuniculi.*

The sequence (177 bp) of the *E. cuniculi* 16S ribosomal RNA gene was deposited in GenBank, Accession number MK615616.

The submitted gene sequence was compared with the aligned sequences available in the NCBI GenBank database. Publicly available 16S rRNA gene sequences for *E. cuniculi* were downloaded from the NCBI GenBank and imported into BioEdit version 7.0.1.4 for multiple alignments using the ClustalW program in BioEdit. A similarity matrix was done using the DNASTAR program (Lasergene, version 8.0). The genetic distance values of species variations of *E. cuniculi* were analyzed with MegAlign project of DNSTAR software.

## Results

### Clinical findings

**Fig. 1:**
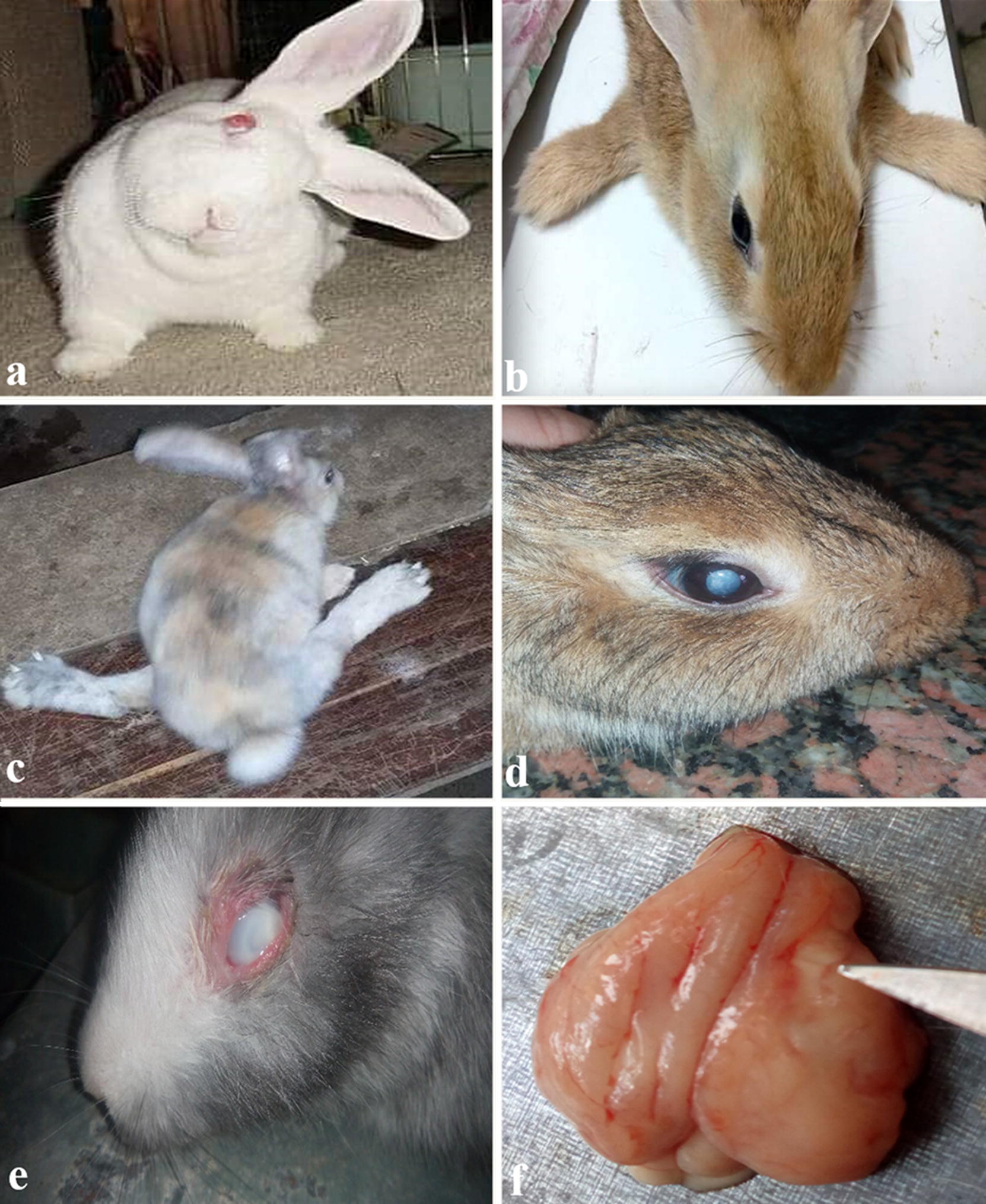
Photographs of clinical and postmortem findings of rabbits infected with *E. cuniculi* showing **a** head tilt in adult rabbit, **b** forelimb paralysis in young rabbit, **c** hind limb paralysis in young rabbit, **d** mild unilateral cataract in right eye, **e** severe unilateral cataract in left eye, and** f** cerebral focal necrosis and congested leptomeninges

Signs of central vestibular disease were observed in 81.5% of the 13,242 rabbits and consisted of ataxia, circling or rotation around the longitudinal axis, head tilt (Fig. 1a) and paresis or paralysis in the forelimbs (Fig. 1b) and hindlimbs (Fig. 1c). Eye lesions, mostly unilateral, were found in 10.5% of the rabbits and were characterized by cataracts and phacoclastic uveitis (Fig. 1d, e). Polyuria, diagnosed by urine scalding of posterior limbs and dehydration, occurred in 2.7% of the rabbits. Additional non-specific clinical signs such as lethargy, anorexia and/or weight loss or a combination of these signs were observed in 5.2% of the rabbits, while 0.26% of the pregnant female rabbits had repeated abortions.

The clinical manifestation varied among animals of different sexes and age groups (Table 1). Morbidity and mortality rates varied among the three farms and between gender and age groups (Tables 2 and 3).

**Table 1:** Incidence of different clinical signs in rabbits infected by *E. cuniculi* during the 4 months of observation (2018)

Rabbits	No. of rabbits	Nervous manifestation head tilt and leg paralysis (%)	Eye lesions uveitis (%)	Polyuria (%)	Combined and non-specific symptoms (%)
Dam	1740	1335 (76.7)	223 (12.8)	77 (4.4)	105 (6.03)
Male	332	250 (75.3)	55 (16.5)	16 (5.1)	11 (3.3)
Young rabbits	11,170	9210 (82.4)	1110 (9.9)	275 (2.4)	575 (5.15)
Total	13,242	10,795 (81.5)	1388 (10.5)	368 (2.7)	691 (5.2)

**Table 2:** Morbidity and mortality rate recorded in adult rabbits during the 4 months of observation (2018)

Farms	Total number of rabbits in each farm^a^	Total no. of investigated rabbits	Dams (> 4 months to 1 year)		Males (> 4 months to 1 year)	
			Morbidity%	Mortality%	Morbidity (%)	Mortality%
A	756	450	370 (48.9)	20 (2.6)	80 (10.6)	0 (0)
B	943	758	650 (68.9)	65 (6.9)	108 (11.4)	1 (0.1)
C	1002	864	720 (71.8)	66 (6.6)	144 (14.37)	0 (0)
Total	2701	2072 (76.7)	1740 (64.4)	151 (5.6)	332 (12.3)	1 (0.037)

^a^Total number of rabbits in each farm including both apparently healthy and clinically affected rabbits

**Table 3:** Morbidity and mortality rate recorded in young rabbits (0 to 4 months) during the 4 months of observation (2018)

Farm	Total number of newly born rabbits	Clinically affected rabbits	Morbidity%	Mortality (%)		
				Total	Early before weaning (PM)^a^	> 2 month (with clinical symptoms)
A	3365	2230	66.3	157 (4.6)	49 (1.5)	108 (3.2)
B	4640	3900	84	780 (16.8)	497 (10.7)	283 (6.1)
C	5694	5040	88.5	756 (13.3)	609 (10.7)	147 (2.6)
Total	13,699	11,170	81.5	1693 (12.3)	1155 (8.4)	538 (3.9)

^a^Without clinical symptoms

### Postmortem findings

Gross pathological changes were observed in 60.7% of the 1171 necropsied rabbits. Cataract and acute focal unilateral uveitis in the anterior chamber were observed in 362 rabbits (Fig. 1d, e). Brain lesions were recorded in 215 rabbits. Meningeal and cerebral vessels were congested with acute severe unilateral multifocal necrosis in the cerebrum (Fig. 1f). Pale enlarged kidneys were found in 84 rabbits with acute mild widespread necrosis of the medulla. Other findings such as endometrial congestion and otitis externa were also observed.

### Histopathological findings

**Fig. 2:**
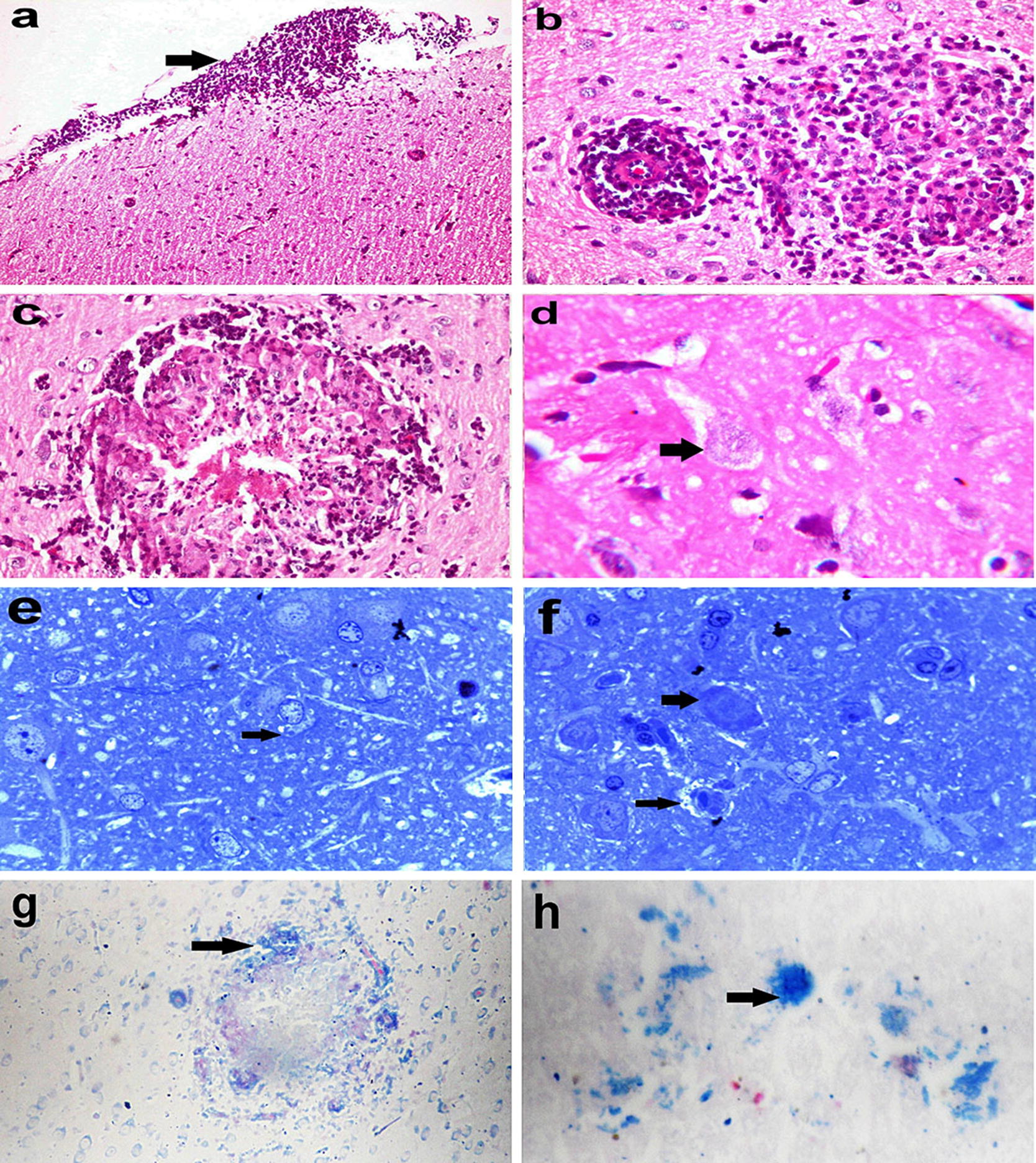
Photomicrographs of *E. cuniculi* in the brain of rabbits. **a** Severe diffuse non-suppurative leptomeningitis and diffuse gliosis (H&E. Obj ×20), **b** perivascular lymphocytic cuff and glial nodule, **c** glial nodule with central necrosis and rod to ovoid-shaped mature *E. cuniculi* spores, and **d** mature *E. cuniculi* spores intracellularly in neurons (H&E, Obj ×40), **e** parasitophorous vacuoles (arrows) and **f** free spores of *E. cuniculi* (arrow) by toluidine blue (Obj ×100), and **g** bluish-stained rod to ovoid-shaped *E. cuniculi* at the periphery of the glial nodule in the brain (Giemsa stain, Obj ×20), **h** liver with bluish-stained rod to ovoid-shaped *E. cuniculi* by Giemsa stain (Obj ×60)

Microscopy of brain sections revealed severe diffuse non-suppurative leptomeningitis (Fig. 2a). In the cerebral cortex and medulla oblongata, multifocal gliosis, perivascular mononuclear cellular cuffing (Fig. 2b) and glial nodules were seen. Some glial nodules had central necrosis (Fig. 2c). Neuronal degeneration and neuronophagia was observed in glial nodules and scattered in the cerebral cortex. Intracellular and extracellular mature spores and pseudocysts that contained basophilic ovoid spores were observed in the cerebral cortex and hippocampus (Fig. 2d). In some rabbits, parasitic spores were found without any associated inflammation. In semithin sections, different stages of the parasite were seen (Fig. 2e, f). Moreover, Giemsa stained ovoid to rod-shaped bluish parasites were observed in neurons (Fig. 2g).

Microscopy of liver sections showed mild degeneration of hepatocytes in the centrilobular area and periportal mononuclear cellular infiltrates. Ovoid to rod-shaped bluish parasites were observed in the liver using Giemsa stain (Fig. 2h).

**Fig. 3:**
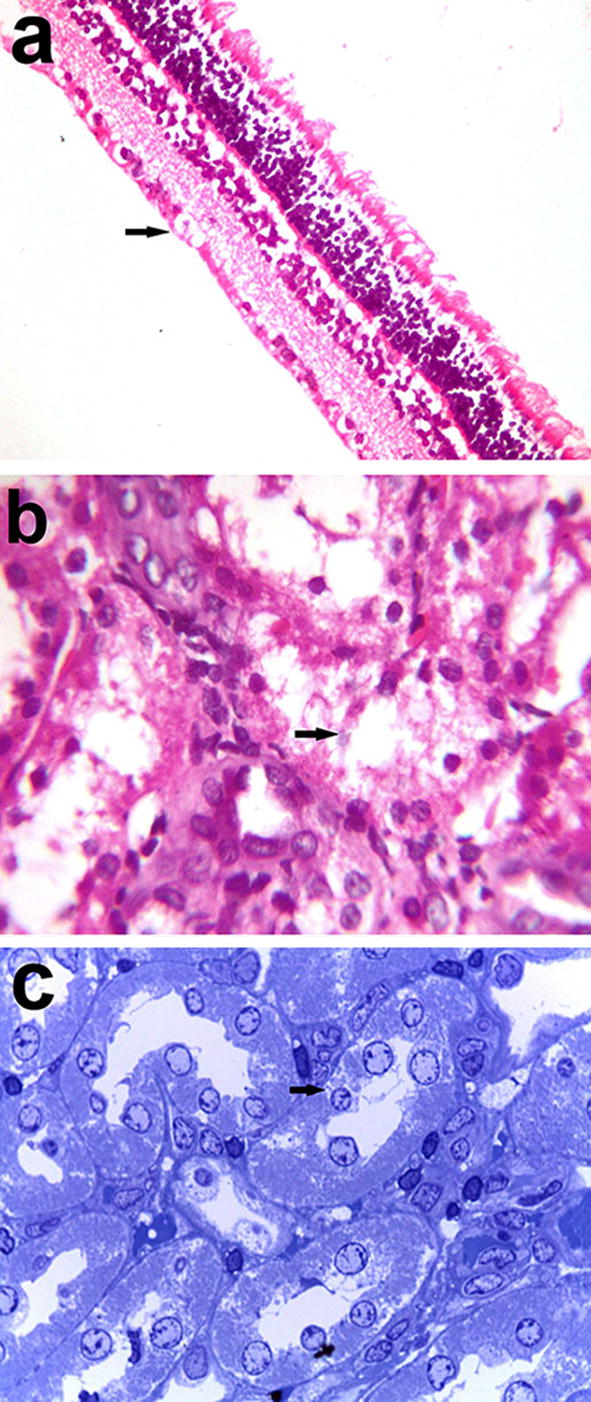
Photomicrographs of the eye and kidney of rabbits infected with *E. cuniculi.* **a** *E. cuniculi* in ganglion cells of the retina (H&E stain, Obj ×40), **b** vacuolation, and necrosis in epithelium of convoluted and collecting tubules of kidney with the presence of small basophilic rod-shaped *E. cuniculi* spores (H&E stain, Obj ×100), and **c** parasitophorous vacuole (arrows) in the renal tubular epithelium by toluidine blue (Obj ×100)

Microscopy of eye sections revealed epithelial ulceration, endothelial necrosis, and oedema of the cornea. The lens capsule was irregular and sometimes had fragmentation of lens fibres and lens epithelial cell necrosis. Infiltration with mononuclear cells was observed in the corneoscleral trabecular meshwork, the junction between the cornea and conjunctiva, iridocorneal drainage angle and in the posterior chamber of the eye admixed with fibrin. The iris was oedematous, infiltrated with scattered mononuclear cells and showed degeneration of the posterior epithelium. The retina was atrophied and detached. Small basophilic rod-shaped parasites were seen in ganglion cells of the retina without associated inflammatory reaction (Fig. 3a).

Microscopy of kidney sections showed vacuolation and necrosis of the epithelium lining the convoluted tubules and collecting ducts. Small basophilic parasite spores (0.6–1.08 × 1.18–1.78 μm) were present inside tubular epithelial cells and were released in the tubular lumen with little or no associated inflammatory reaction (Fig. 3b). In semi-thin sections, cysts containing ovoid parasitic spores were observed in the renal tubular epithelium with the displacement of the nuclei towards their apical surface (Fig. 3c). The spores were released from the apical surface of the tubular epithelium that showed pyknosis or karyolysis.

### TEM

**Fig. 4:**
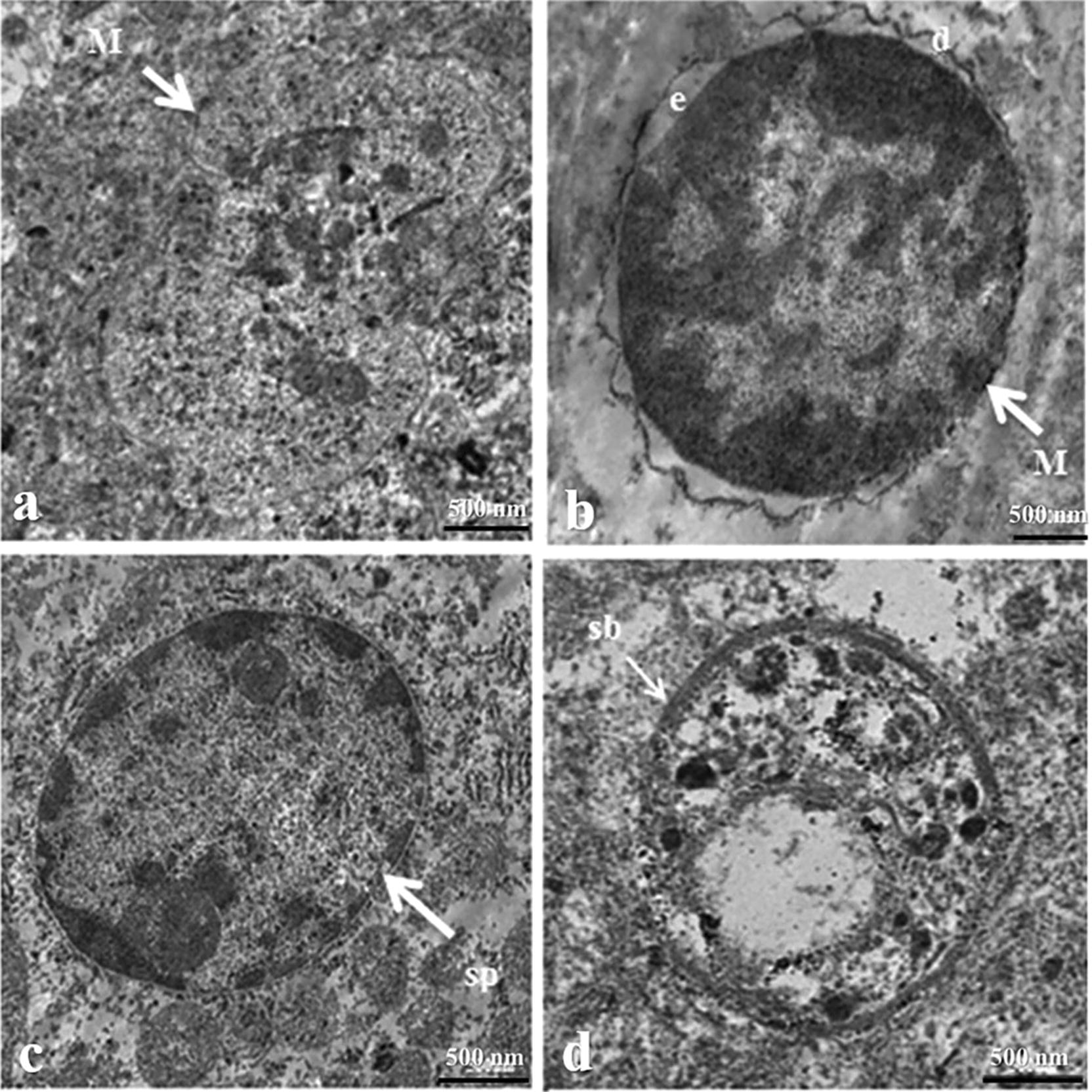
Transmission electron micrographs of different stages of *E. cuniculi* in brain tissue from infected rabbits showing **a**, **b** meront of different shape, **c** sporont, and **d** sporoblast (*e* electron-lucent endospore layer, *d* dense outer coat, *M* meront, *sb* sporoblast, *sp* sporont)

The meront, sporont, and sporoblast stages of *E. cuniculi* were all found in brain sections. The meront stage was ovoid with a size of 2.21–3.1 × 3.175–3.8 μm, had an electron-lucent endospore layer and was surrounded by a dense outer coat. The sporont and sporoblast stages were also ovoid but measured only 1.91 × 2.3 μm and 1.85 × 2.2 μm, respectively (Fig. 4).

**Fig. 5:**
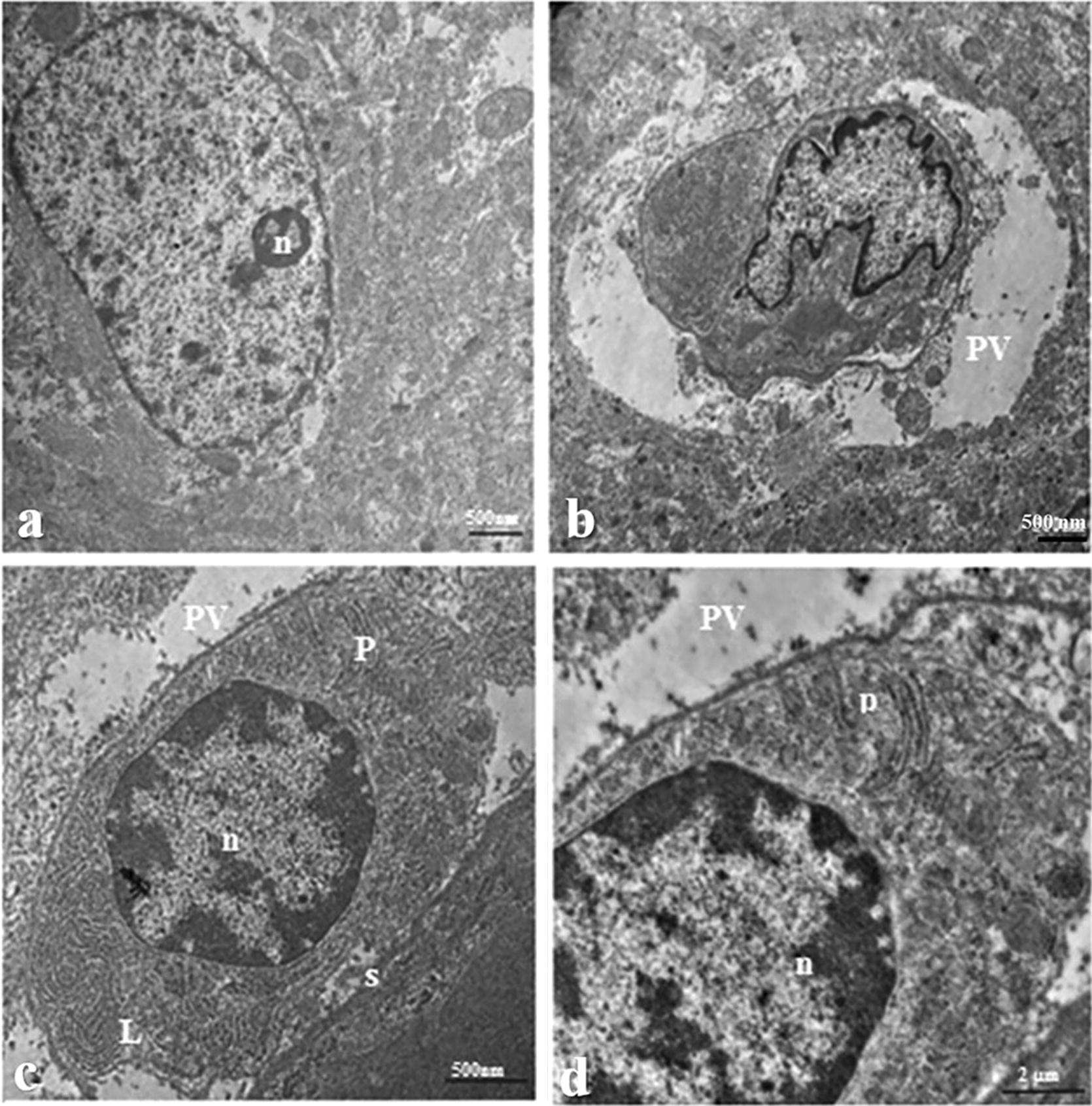
Transmission electron micrographs of two stages of *E. cuniculi* in kidney tissue from infected rabbits showing **a**,** b** sporont, **c** longitudinal section through mature spore demonstrating lamellar polaroplast and sporal wall, and **d** spore with coils of polar tube in a single row (n = 5) (*L* lamellar polaroplast, *n* nucleus, *P* polar tube, *PV* parasitophorous vacuole, *S* sporal wall)

Only the sporont stage and mature spores were detected in the kidney. They had a characteristic ovoid shape. The mature spores had lamellar polaroplast, with five single rows of coiled polar tubes surrounded by spore walls. The size of sporonts was 2.5–2.55 × 4.2–3.85 μm, while a mature spore measured 1.75 × 3.3 μm (Fig. 5). *E. cuniculi* were found intracellularly and had different stages with different size located in the brain and kidney.

### Molecular findings

All examined brain, kidney, eye and liver tissues were positive for the expected amplicon molecular weight (260 bp). The sequence analyses from the ten selected positive samples were identical and revealed a 100% similarity to *E. cuniculi* strains deposited in the GenBank database.

**Fig. 6:**
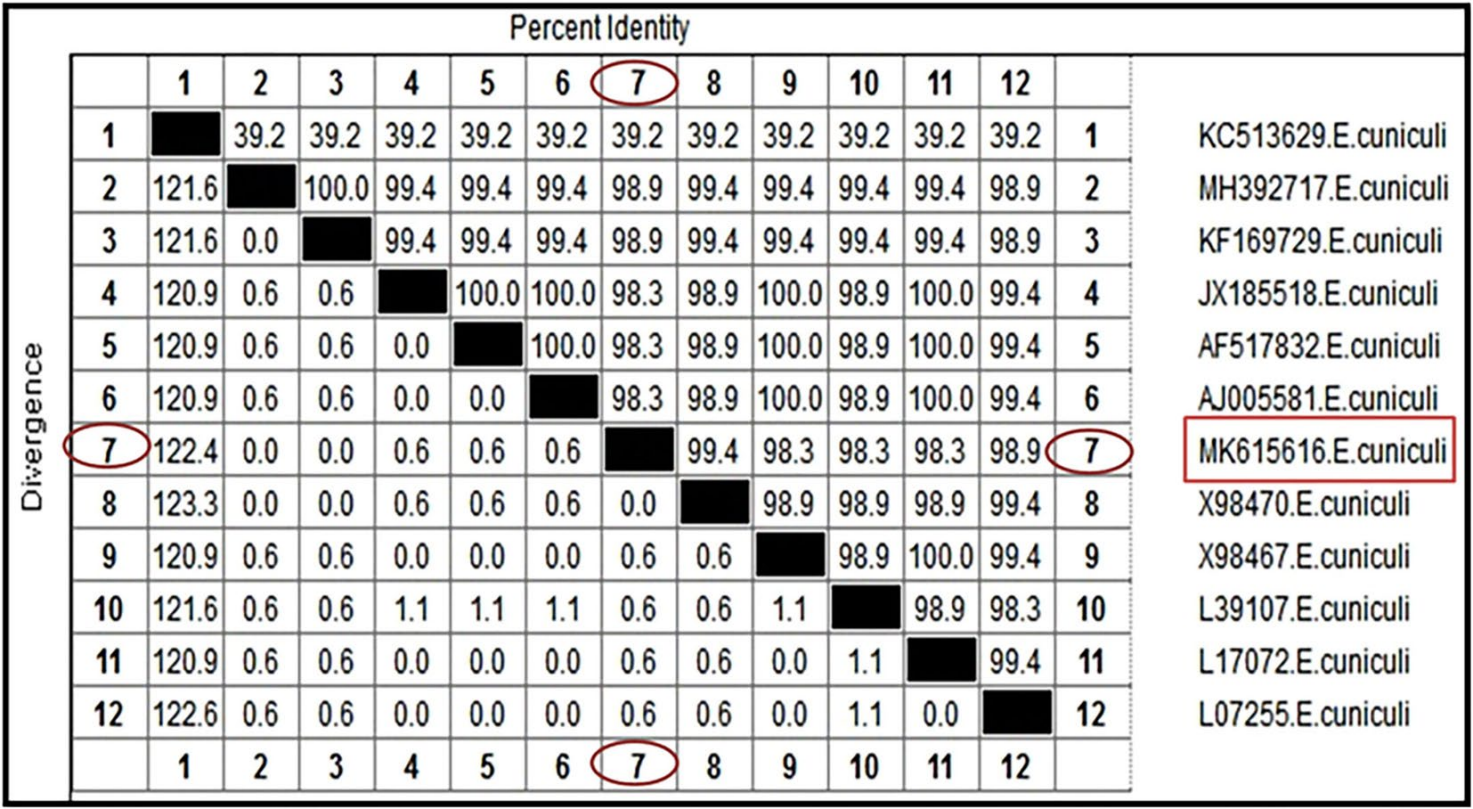
Similarity (percent identity) and genetic divergence of 16S ribosomal RNA sequences of *E. cuniculi* from a rabbit in Egypt (representing number, 7) as compared with the most similar reference sequences (GenBank). The 16s ribosomal RNA sequenced in this study is marked and represented as number, 7

Inter- and intra-species analyses of genetic distance among the obtained sequence and other 11 aligned sequences available in the NCBI GenBank database of *E. cuniculi* were performed. (Fig. 6). The genetic identity of *E. cuniculi* had a high sequence homology (99.4% similarity) with the Donovan sequence of *E. cuniculi* (Accession number X98470) isolated from a human in the UK (Fig. 6). Interspecies analysis based on the genetic distance values indicated a zero level of genetic divergence (GD) within the genospecies of *E. cuniculi* isolated from a human (Donovan sequence). However, the Donovan sequence of *E. cuniculi* was genetically more distant (GD 0.6) from *E. cuniculi* isolated from humans from the UK and USA (Accession numbers (X98467, L39107, L17072, and L7255).

## Discussion

The rabbit industry in Egypt is at present flourishing and rabbits have become an alternative source for meat [24]. The transmission of *E. cuniculi* to rabbit farms threatens the fate of this industry due to high morbidity and a potential zoonotic risk [7]. The high morbidity of the disease in the three investigated farms indicates that this parasite was endemic in the farms. The high rate of infection may be due to the ingestion of contaminated food or water with *E. cuniculi* spores further spread through the urine of infected rabbits [12]. The difference in morbidity in the three investigated farms might be due to environmental factors and husbandry. Females had a higher morbidity rate (64.4%) than males (12.3%), which is contradictory to previous findings [31]. The highest morbidity rate was recorded in the young age group (81.5%), which was similar to previous findings [31].

The diagnosis of diseases causing nervous signs in rabbits are challenging as they may be caused by many conditions. In the present study, the association of nervous signs with ocular lesions suggested encephalitozoonosis. Nervous signs including central vestibular disease and unilateral ocular lesions are the main signs associated with *E. cuniculi* infection in rabbits [32–35] and leptomeningitis and necrotic foci in the brain are known as the main lesions associated with *E. cuniculi* [6] as also observed in the present study. The necrotic foci were mainly glial nodules with central necrosis as reported previously [36]. Brain lesions varied in severity between rabbits. The formation of glial nodules might be due to the chitin of endospore microsporidians as chitin is chemotactic for macrophages and results in the recruitment of CD8+ cells, production of IL-2 and IFN-γ, which eventually lead to formation of granulomas [14, 37, 38].

Ocular lesions due to parasitic colonisation of the lens are very common in rabbits having encephalitozoonosis [15, 39, 40]. The lens capsule may rupture due to disruption of the fibres causing phacoclastic uveitis and zonal granulomatous lens uveitis, as well as cataracts [41]. Most previous studies have reported that the posterior parts of the eye, including the vitreous body, retina, and choroid, are unaffected [42]; however, in the current study, the parasite was observed in the retina.

Some rabbits showed symptoms of polyuria and urinary incontinence. *E. cuniculi* infection results in renal interstitial lymphocytic proliferation, necrosis of tubular epithelium, and interstitial fibrosis associated with presence of intact or ruptured parasitic cysts [17]. In the present study, renal tissue response was not obvious, although spores and degeneration of tubular epithelial cells were observed.

Hepatic lesions were restricted to mild periportal mononuclear inflammatory cell infiltration and mild vacuolar degeneration but the parasite was observed by Giemsa stained sections. In contrast, other studies have reported moderate to severe lesions in the liver, including periportal nonsuppurative interstitial infiltration, fibrosis and necrosis of the periportal area [17].

To the best of our knowledge, the ultrastructure of *E. cuniculi* stages in the brain and kidneys of rabbits has not been reported previously. The different intracellular stages of *E. cuniculi* can be discriminated from each other because the parasite undergoes morphological changes [43]. The mature spores of *E. cuniculi* were found to have a nucleus, lamellar polaroplast, a single row of coiled polar tubes and surrounding spore wall similar to the findings of Vavra and Larsson [44], and Habenbacher et al. [45]. The mature spore of *E. cuniculi* ranged from 1.75 × 3.3 μm, whereas the spore of *Encephalitozoon* spp. in another study was 1.0–1.5 × 2.0–2.5 μm [9]. The variation between the measurements might be attributed to the tissue type or the stage of parasite development during sample collection and the host [43]. The main feature of differentiation of *E. cuniculi* from all other *Encephalitozoon* spp. is the parasitophorous vacuole (PV). *E. cuniculi* is the only species of microsporidia known to exist in a septated PV that separates each developing spore [46].

Molecular detection of the *Encephalitozoon* spp*.* was done by amplification of the specific 16*S* gene [30]*.* The results confirmed *E. cuniculi* infection in all examined brain, kidney, eye and liver tissues. This result is in accordance with other studies that have suggested that encephalitozoonosis is a systemic disease affecting many organs [47]. The identity matrix for 16S rRNA for *E. cuniculi* presented 99.4% similarity with* E. cuniculi* (Donovan sequence) isolated from human thus indicating a potential zoonotic transmission of *E. cuniculi* circulating in rabbits farms in Egypt.

## Conclusions

Encephalitozoonosis in farmed rabbits was very prevalent in three farms investigated in middle Egypt and associated with a high mortality/culling rate. Sequence analyses revealed a potential zoonotic risk of this infection in Egypt.

## Data Availability

The datasets used and analysed during the current study are available from the corresponding author on reasonable request. The sequence of the *E. cuniculi* 16S ribosomal RNA gene was deposited in GenBank, Accession number MK615616.
